# Intestinal Lesions Due to Rhodococcus equi in a Patient With Advanced Retroviral Disease and Pulmonary Infection: A Case of Colonic Malakoplakia

**DOI:** 10.7759/cureus.42248

**Published:** 2023-07-21

**Authors:** Paola Di Carlo, Luca Pipitò, Elisabetta Orlando, Sandro Bellavia, Giovanni Boncori, Caterina Sarno, Vito Rodolico, Teresa Fasciana, Consolato Sergi, Antonio Cascio

**Affiliations:** 1 Department of Infectious and Tropical Diseases, Azienda Ospedaliera Universitaria Policlinico (AOUP) "Paolo Giaccone", Palermo, ITA; 2 Department of Health Promotion, Mother and Child Care, Internal Medicine and Medical Specialties, "G. D'Alessandro", University of Palermo, Palermo, ITA; 3 Department of Pathology, Azienda Ospedaliero Universitaria "Paolo Giaccone", Palermo, ITA; 4 Department of Health Promotion, Mother and Child Care, Internal Medicine and Medical Specialties,, "G. D'Alessandro", University of Palermo, Palermo, ITA; 5 Department of Radiological Sciences, Azienda Ospedaliero Universitaria "Paolo Giaccone", Palermo, ITA; 6 Department of Microbiology and Virology, Azienda Ospedaliero Universitaria "Paolo Giaccone", Palermo, ITA; 7 Department of Medicine and Pathology - Laboratory, Children’s Hospital of Eastern Ontario, University of Ottawa, Ottawa, CAN

**Keywords:** von kossa special stain, differential diagnosis, colonic lesions, colonic malakoplakia, hiv aids, r. equi

## Abstract

In humans, *Rhodococcus equi *(*R. equi*) is a zoonotic infection usually involving immunocompromised subjects, only rarely affecting immunocompetent subjects. Herein, we describe an *R. equi *infection in a 50-year-old Russian man with acquired immune deficiency syndrome (AIDS) who presented with pulmonary cavitary lesions and clinical manifestation of colonic malakoplakia. A colonoscopy examination showed ulceration and mucosal erosion, and the histological findings confirmed the colonic malakoplakia. The patient recovered from pulmonary and gastrointestinal disease after four weeks of antibiotic treatment with intravenous ciprofloxacin and oral azithromycin and also underwent subsequent long-term oral antibiotic treatment to achieve clinical and immune restoration after antiretroviral therapy. Infectious disease pathology subspecialties should always consider *R. equi* chronic infection as a cause of malakoplakia in patients with AIDS. As only a few cases of colonic malakoplakia associated with *R. equi *are reported in the literature, these cases are important to describe, especially for clinical and treatment management.

## Introduction

Malakoplakia is a rare granulomatous inflammatory disease infection, referred to as a type of tumor-related disease, which mainly affects immunocompromised people but can also affect healthy patients [1,2]. Patients who are severely immunocompromised, including those with advanced retroviral disease, can present with gastrointestinal manifestations that have a broad differential diagnosis. These include opportunistic infections such as cytomegalovirus colitis, Mycobacterium avium complex infection, gastrointestinal malignancies, non-Hodgkin lymphoma, Kaposi's sarcoma, and anal cancer, and other bacterial, viral, and parasitic infection [3]. Historically, *E. coli* has been reported to be among the possible infectious agents of malakoplakia, but other microorganisms have also been reported to be involved, although often the cause remains unknown [2,4,5]. Thus, although uncertainty remains regarding the specific pathogenesis of malakoplakia, it seems to be associated with the defective intracellular killing of ingested microorganisms by macrophages. It is evident that among the infectious agents, all those mycobacteria that stimulate a granulocyte response are protagonists, especially when the macrophages are involved in the inflammatory response [6]. *R. equi* pulmonary malakoplakia is reported in both human immunodeficiency virus (HIV)-positive and HIV-negative subjects, although this pathogen is known as an opportunistic infection in AIDS patients [2,7-9]. Similar to mycobacteria, *R. equi* is an intracellular pathogen, entering the phagocytes within host macrophages [10]. Although cases of colonic *R. equi* associated with malakoplakia have been rarely reported in AIDS patients, gastrointestinal and colorectal malakoplakia disease is a condition involving both immunocompromised and healthy subjects [11-13,14]. Our case report illustrates colonic malakoplakia in a patient with *R. equi* bacteremia and pulmonary disease.

## Case presentation

A 50-year-old Russian male patient from a rural area of Sicily with chronic hepatitis C virus infection visited our Infectious Disease Department in February 2021 with complaints mainly of altered mental status and an AIDS late-presenter diagnosis. His history revealed that he moved to Italy about a year prior; he is a heavy smoker and had worked in the agriculture sector in Russia and Italy, where he came into contact with farm animals and horses.

**Figure 1:**
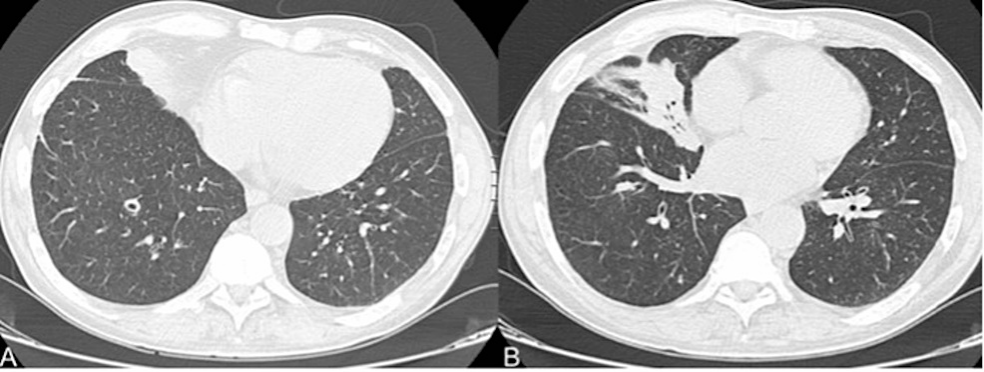
Pulmonary cavitary nodule on computed tomography in the lateral basal segment of the right lower lobe (A); parenchymal consolidation with aerial bronchogram in the medial segment of the middle lobe (B).

On admission, he was afebrile, and his vital signs were as follows: blood pressure of 100/70 mmHg; heart rate of 100 beats per minute; 92% blood oxygen saturation in room air; respiratory rate of 22 breaths per minute; Glasgow Coma Scale score of 9; and a Numeric Rating Scale pain score of 3. The physical examination findings showed notably reduced breath sound in all lung fields and a painful abdomen upon superficial and deep palpation, especially in the epigastrium, right hypochondrium, and right flank. His laboratory results revealed microcytic anemia (Hgb 8.2 g/dL, MCV 77.5 fl), hyponatremia (132 mmol/L), C-reactive protein levels of 43.04 mg/L, a CD4+ T-lymphocyte count of 5 cells/mm3, an HIV-1 viral load (VL) of 1,770,000 copies/ml (cp/ml), and an HCV viral load of 4,150 UI/ml. Total-body computed tomography showed cerebral white matter damage consistent with mild-to-moderate cerebrovascular insult, a cavitary nodule in the lateral basal segment of the right lower lobe, and a parenchymal consolidation in the medial segment of the middle lobe in the right lung (Figure 1).

Bronchoalveolar lavage (BAL) and fluid cytology showed a typical cell pattern with no indications of malignancy. Likewise, BAL fluid microbiological investigation for aerobic, anaerobic, mycobacteria, and yeast, microscopy and culture, and tests for *Pneumocystis jirovecii*, Aspergillus spp, SARS-CoV-2, and other viruses returned as negative. We started antiretroviral therapy (ARV) consisting of bictegravir (50 mg) combined with emtricitabine (200mg) and tenofovir alafenamide (25 mg) once daily, as well as prophylaxis with trimethoprim/ sulfamethoxazole 160/800mg once daily, to prevent opportunistic diseases​ such as ​​​​​​*Toxoplasma gondii* and *Pneumocystis jirovecii*. The BAL and blood sample culture showed growth of *R. equi*. The strain was identified at the microbiological Department of Microbiology, Policlinico Paolo Giaccone Hospital, by the automated MB/BacT system (bioMérieux, Marcy-l'Étoile, France) and subjected to mycobacteria identification by phenotypic methods and Accuprobe® (Gen-Probe, San Diego, CA, US). It showed susceptibility to ciprofloxacin, clarithromycin, chloramphenicol, gentamicin, teicoplanin, and trimethoprim/ sulfamethoxazole and resistance to penicillin and tetracycline according to the EUCAST criteria [15].

**Figure 2:**
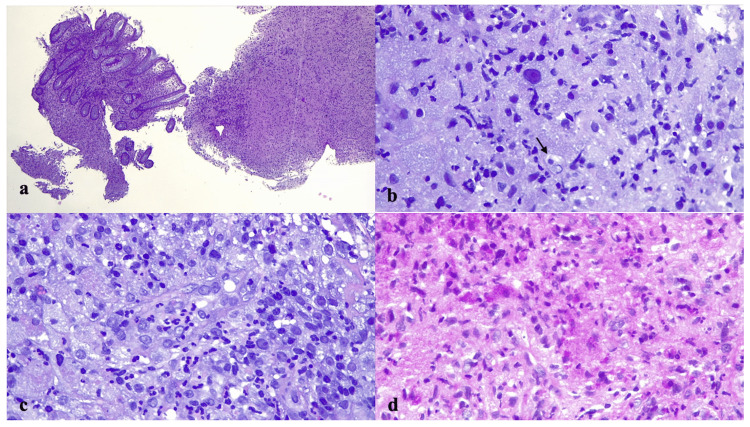
Histological section. (a) Hematoxylin & eosin (HE) stain x 40 magnification: Intestinal biopsy with extensive ulcerative lesion associated with inflammatory infiltrates; (b) HE stain x 200 magnification: Foamy histiocytic infiltrate and rare nucleomegaly cells with inclusion-like nucleoli resembling an "owl's eye" appearance (arrow); (c) HE stain x 200 magnification: Mixed inflammatory infiltrate with foamy histocytes, lymphocytes, and polymorphonuclear cells. (d) PAS stain x 200: Histocyte infiltrate with PAS-positive intracytoplasmic coccobacilli.

We started ciprofloxacin 400 mg/12 hours IV therapy and azithromycin 250 mg/day oral treatment. After six days of antibiotic treatment, our patient experienced anemia and bloody diarrhea and, hence, considering his treatment-naïve status, we ordered an esophagogastroduodenoscopy and colonoscopy immediately. The colonoscopy showed ulcerative lesions on the entire surface of the colon, and some tracts of colonic mucosa showed a slight elevation of the mucosal surface. The intestinal biopsies showed extensive erosive-ulcerative phenomena of the surface epithelium with partial distortion and sometimes destruction of the glandular crypts and intense lymphohistiocytic and neutrophilic granulocytic infiltration of the lamina propria. Numerous macrophages were seen in the chorion and submucosa, as better revealed by the specific histiocytic marker CD68, and contained intracytoplasmic coccobacilli that were positive upon PAS staining; they sometimes showed foamy cytoplasm (Figure 2).

**Figure 3:**
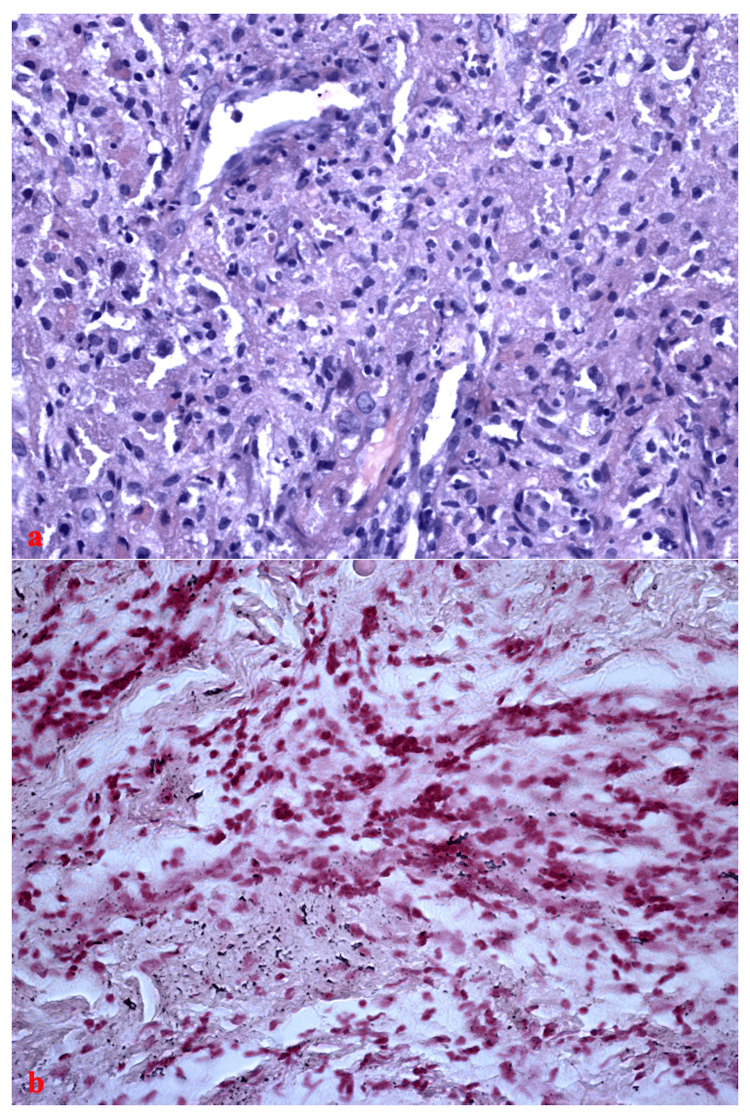
Histological section. (a) HE stain x 200 magnification: intracytoplasmic Michaelis-Gutman bodies are present in the colonic biopsy; (b)Von Kossa special stain x 200 of colonic biopsy highlights intracytoplasmic Michaelis-Gutman bodies characterized by calcium accumulation.

The PAS-positive material detected in the macrophages was homogeneous, finely stippled, or granular-shaped. Neither the Ziehl-Neelsen acid-fast stain nor the Giemsa stain showed any microorganisms. A more careful analysis of the hematoxylin-eosin stain showed intracytoplasmic Michaelis-Gutman bodies, and the Von Kossa special stain confirmed it (Figure 3).

The histological findings were suggestive of colonic malakoplakia associated with a microorganism infection. Considering the *R. equi *strain isolated from the BAL and blood cultures, our infectious disease pathology has been regarded as colonic malakoplakia associated with *R. equi* chronic infection. The patient was discharged after one month of hospitalization to return home with home health care and continued to receive azithromycin, levofloxacin, bictegravir, emtricitabine, and tenofovir alafenamide fumarate. The patient's symptoms improved, and no recurrence of pulmonary and colonic involvement was identified during the 12 months of treatment with azithromycin (250 mg daily) and levofloxacin (750 mg daily). The follow-up colonoscopy showed a resolution of the previous lesions. The patient showed regularity in attending hospital follow-up appointments. In May 2022, his CD4+ T-lymphocyte count was 184 cells/mm3 and his HIV-1 viral load (VL) was 81.4 cp/ml. In August 2022, no symptoms of recurrence were detected.

## Discussion

Our case report describes a case of colonic malakoplakia associated with *R. equi* infection in a treatment-naïve HIV-positive patient with severe immunodepression who showed bacteremia, and lung and colonic lesions. Diagnosis may be challenging with the macroscopic aspect of the colonic lesion resembling colonic cancer and lung lesions suggesting tuberculosis.

*R. equi* is a veterinary pathogen, originally isolated from horse lungs, that can cause substantial morbidity in patients who are both immunocompromised and occupationally or recreationally exposed to livestock and farming environments [16]. As a human facultative intracellular bacterium, *R. equi* survives and multiplies in macrophages and establishes its specific niche inside the host cell with the ability to survive in macrophages, probably due to inadequate acidification of phagolysosomes [17,18]. Pulmonary involvement is a predominant feature in *R. equi* infection and the most common radiographic changes on CT are ill-defined consolidation and irregular areas of cavitation in the lungs with preference for the upper lobes [3].

If the pulmonary localization of *R. equi* associated with malakoplakia is well known, gastrointestinal and colorectal localization is rarely reported [16]. In our case, the intestinal biopsies showed histological findings of malakoplakia. The colonic chorion and submucosa had numerous macrophages with foamy cytoplasm containing countless intracytoplasmic granules that were positive upon PAS staining. Furthermore, Michaelis-Gutman bodies were highlighted by Von Kossa special stain. The histiocytic elements were negative for the Ziehl-Neelsen and Giemsa stains [16,17]. We also found extensive erosive-ulcerative phenomena of the surface epithelium with partial distortion and destruction of the glandular crypts by intense lymphohistiocytic and neutrophilic granulocytic infiltration of the lamina propria. Endoscopic biopsies of the gut of an immunocompromised patient revealing the presence of foamy macrophages with intracellular PAS-positive granules should make one suspicious of a possible *R. equi *infection. The accumulation of foamy macrophages can also be seen in other infections such as *Mycobacterium tuberculosis* and, recently, in inflammation in atherosclerosis and cancer [18-20].

Moreover, malakoplakia should also be considered in differential diagnoses along with other opportunistic colonic diseases, especially Kaposi's sarcoma, tuberculosis, and/or atypical mycobacterial infection. In our case report, a suspected colonic infection due to *R. equi* was suggested early by the identification of the pathogens in both the BAL and blood culture. In these cases, the opportunistic pathogen can spread, and clinicians must pay attention to other symptoms and signs evocative of disseminated disease. *R. equi* enter colonic macrophages and tissue-resident macrophages, which causes a granulomatous inflammatory response, and colonic malakoplakia lesions occur due to the macrophages being unable to completely destroy the bacteria that they have phagocytosed. Michaelis and Gutmann identified the malakoplakia condition based on the Michaelis-Gutmann bodies in 1902, which are intracytoplasmic calcospherules pathognomonic for malakoplakia. Calcium accumulation is identified by Von Kossa special stain. However, this feature may be not essential, especially in the early phase of the disease [21-23].

In immunocompromised patients, histology is essential, not only to diagnose malakoplakia but also to establish the role of opportunistic pathogens and start an adequate approach to treatment, such as antibiotic therapy [24,25]. The emergence of resistant strains of *R. equi* could complicate the clearance of this pathogen both in localized and disseminated infections, especially in immunocompromised subjects or subjects with chronic disorders such as diabetes [26]. In addition, *R. equi* infections can be challenging due to their tendency to form biofilms and have antibiotic resistance [26,27]. Clinical isolates in the United States (US) are usually susceptible to trimethoprim/ sulfamethoxazole, whereas more than half of the isolates in Europe are resistant [28]. This difference in susceptibility to trimethoprim/ sulfamethoxazole may suggest that the US and Europe have different strains of *R. equi*. It is unclear why and how the bacterium in Europe acquired resistance to trimethoprim/ sulfamethoxazole. It is possible that differences in the usage of antibiotics in domestic animals between these two continents have resulted in the disparity in susceptibility to the antibiotic. However, in our case, the isolate was susceptible to trimethoprim/ sulfamethoxazole. An empiric antibiotic treatment regimen for *R. equi* infection would include two to three intravenous agents (vancomycin, imipenem, clarithromycin/ azithromycin, rifampin, an aminoglycoside, ciprofloxacin/ levofloxacin, or trimethoprim/ sulfamethoxazole), administered for at least two weeks if the patient is immunocompromised, or two oral agents (clarithromycin/ azithromycin, rifampin, ciprofloxacin/ levofloxacin, or cotrimoxazole) if the patient is immunocompetent with mild or moderate disease. In our case, we administered only intravenous ciprofloxacin associated with oral azithromycin for one month and after oral therapy with azithromycin and levofloxacin with a good outcome. Despite colonic surgery being necessary in another case described in the literature [11], our patient had a full recovery with only long-term antimicrobial therapy without intestinal complications. 

## Conclusions

In conclusion, *R. equi* should be considered in the differential diagnosis of intestinal lesions in AIDS patients and as an agent of malakoplakia. Endoscopic investigation on admission in all immunocompromised patients with unspecified gastrointestinal symptoms is essential for opportunistic infection diagnosis, and collaboration with pathologists is mandatory to confirm the role of opportunistic pathogens in these cases. Although the necessity of surgery and two intravenous antibiotics are reported in the literature for the management of *R. equi* infections, especially in cases of malakoplakia in immunosuppressed patients, we observed a resolution of the clinical picture with only one intravenous agent associated with oral therapy, and subsequentially long-term oral antibiotic therapy, without recurrences.
